# The Hospital Drug Supply

**Published:** 1916-07-29

**Authors:** 


### THE HOSPITAL DRUG SUPPLY.

### A Word of Caution to Buyers.

Some months ago in The Hospital it was shown how a change had come over the conditions ruling in the drug market, and how necessary it was for buyers of pharmaceutical supplies for hospitals to act with caution. Up to that time there had been a constant advance in the prices of many of the most commonly prescribed drugs ever since the beginning of the war, and on occasion the advance had been violent. The values of drugs had, in fact, risen to extraordinary and, in some cases, fantastic levels, but, observing a significant undercurrent, we deemed it necessary to advise hospital authorities of the necessity, in the interests of economy, to watch the market carefully, and to make purchases only after considering the market conditions of any given drug. That article appeared at an opportune moment, and if the hints that were therein given have been acted upon there must have been a substantial saving in the drug bill at many institutions. Ever since that time, when the curious undercurrent was hardly observable, there has been a steady downward tendency in the values of costly drugs, and it would be well to consider whether this tendency is likely to continue.

### Bromides and Salicylates.

The fabulous prices to which potassium bromide, ammonium bromide, salicylic acid, and sodium salicylate had risen necessitated a heavy drain on institutional funds. Any buyer who purchased in quantities supplies of these drugs when they had reached the remarkable figures at which they were quoted when the article in question appeared has suffered the disconcerting experience of observing prices gradually dwindle, until potassium bromide is now worth less than one-third its value a few months ago, and salicylic acid is also almost half its value. One of the main causes of the decline in the prices of bromides is the increased production in the United States, and another is the "nervousness" of holders of stocks occasioned by optimistic rumours on the other side of the Atlantic concerning the progress of the war. With regard to salicylates, the steady week-by-week fall in prices has been due not only to increased production in America, but to the fact that British makers have succeeded in producing an improved quality of drug, and improving the extent of their output; indeed, after the war it seems probable that we shall not be under the obligation of going to Germany for our salicylates. The price of salicylic acid, it is true, is still ten or eleven times the pre-war figure, but a few months ago it was twenty times. After the war, when the raw material is no longer required for the manufacture of explosives, and when carbolic acid is obtainable at normal figures, we should be able to produce salicylates in this country in competition with the German makers, provided steps are taken—as no doubt they will be—to prevent dumping designed to kill a new British industry.

What about the immediate future of the market for bromides and salicylates? To give a definite answer to this question is impossible, but it seems most improbable that we shall see a reversion to the exorbitant quotations which ruled a short time ago.

### Synthetic Drugs.

Synthetic drugs generally have not followed the same downward course as salicylic acid, although with one or two exceptions there is undoubtedly an easier tone in the market. Acetylsalicylic acid has maintained its price fairly well in face of the continued strong demand, but in many quarters the opinion is held that any change in value would not be in an upward direction; the output of this drug has undoubtedly improved, but there are still inferior samples on the market, and the buyer should therefore be cautious. Acetanilide also shows signs of receding in value, but the recession is not likely to be very violent. Such drugs as barbitone, sulphonal, and trional have not varied greatly in price of late, and nobody is buying more than necessary to cover immediate consumption needs. On the other hand phenacetin has advanced, and at the present moment is difficult to obtain. Similarly, it is difficult to buy phenolphthalein at any price, and it will soon be possible to say the same of resorcin.

### Other Drugs.

Cod-liver oil has been steadily tending downwards in price for some time past, but even now the figure is so much above the normal that buyers will only make such purchases as are absolutely necessary, expecting that the downward movement has not yet reached its limit. Castor oil is also among the drugs that are cheaper, and it is not improbable that the value will recede still further. The market position of opium has changed but little during the past few months, and as the supply seems to be adequate there would appear to be no reason to expect any higher prices in the immediate future; indeed, any change might be in the opposite direction. Makers of morphine and codeine are less busy than they were, and here again no higher prices are looked for. Quinine has been a very dull market for some long time, and the usual Continental brands are obtainable at several pence per ounce below the price quoted a month or so back, and there seems to be no reason for haste in making purchases. On the whole the best advice to give to buyers of hospital drugs is that they should continue to act with great caution, and before buying ahead of immediate requirements make a thorough inquiry into the market condition of each drug required. This advice is, of course, good at all times, but at the present moment it is especially applicable.

